# Involving Medical Students in Providing Patient Education for Real Patients: A Scoping Review

**DOI:** 10.1007/s11606-017-4065-3

**Published:** 2017-06-09

**Authors:** Thomas W. Vijn, Cornelia R. M. G. Fluit, Jan A. M. Kremer, Thimpe Beune, Marjan J. Faber, Hub Wollersheim

**Affiliations:** 1 Radboud University Medical Center, Radboud Institute for Health Sciences, Scientific Center for Quality of Healthcare, Nijmegen, The Netherlands; 2 Radboud University Medical Center, Radboudumc Health Academy, Department for Research in Learning and Education, Nijmegen, The Netherlands

**Keywords:** patient education, medical education, transfer learning, quality of care, scoping review

## Abstract

**Background:**

Studies suggest that involving students in patient education can contribute to the quality of care and medical education. Interventions and outcomes in this field, however, have not yet been systematically reviewed. The authors examined the scientific literature for studies on interventions and outcomes of student-provided patient education.

**Methods:**

Four databases (MEDLINE, EMBASE, ERIC, PsycINFO) were searched for studies reporting patient education, undergraduate medical students, and outcomes of patient education, published between January 1990 and October 2015. Facilitators of and barriers to educational interventions were assessed using the Learning Transfer System Inventory. The learning yield, impact on quality of care, and practical feasibility of the interventions were rated by patients, care professionals, researchers, and education professionals.

**Results:**

The search resulted in 4991 hits. Eighteen studies were included in the final synthesis. Studies suggested that student-provided patient education improved patients’ health knowledge, attitude, and behavior (nine studies), disease management (three studies), medication adherence (one study), and shared decision-making (one study). In addition, involving students in patient education was reported to enhance students’ patient education self-efficacy (four studies), skills (two studies), and behavior (one study), their relationships with patients (two studies), and communication skills (two studies).

**Discussion:**

Our findings suggest that student-provided patient education—specifically, student-run patient education clinics, student-provided outreach programs, student health coaching, and clerkships on patient education—has the potential to improve quality of care and medical education. To enhance the learning effectiveness and quality of student-provided patient education, factors including professional roles for students, training preparation, constructive supervision, peer support on organizational and individual levels, and learning aids should be taken into account. Future research should focus on further investigating the effects found in this study with high-level evidence.

## INTRODUCTION

Healthcare is shifting towards person-centered care and patient empowerment, and physician–patient relations are evolving towards shared decision-making.^1^ Part of the effort to improve care outcomes involves educating patients with regard to disease and treatment processes through the use of various approaches aimed at improving self-care,^2^ health literacy,^3^ treatment adherence,^4^ and health outcomes.^5^^–^ 8 Along with patient empowerment, medical education is shifting towards professional roles for students in patient care.^9^ Undergraduate students are progressively involved in care practice during longitudinal clerkships,^10^^,^ 11 in service-learning education,^12^^,^ 13 and in student-run clinics.^14^^,^ 15 To enable medical students to play a more substantial and meaningful role in care practice, new educational strategies need to be explored.^9^

Workplace learning among medical students in care practice at an early stage of medical education enhances students’ professional identity and attitude, team experience and skills, and their ability to perform tasks.^16^ Moreover, student-provided patient education is hypothesized to benefit both patients and students.^17^ Various examples have been reported on involving undergraduate healthcare students in providing health promotion interventions.^18^^,^ 19

Despite the above-mentioned examples and effects, no reviews have yet systematically assessed the specific interventions and outcomes that have been reported when undergraduate medical students are involved in patient education. Our scoping review examines the scientific literature for studies on interventions and outcomes of student-provided patient education and evaluates ways in which these interventions can benefit patient care and medical education.

## METHODS

We searched four databases (MEDLINE, EMBASE, ERIC, PsycINFO) for studies on student-provided patient education, published between January 1990 and October 2015, using Preferred Reporting Items for Systematic Reviews and Meta-Analyses (PRISMA) guidelines.

The search strategy combined two themes: patient education and undergraduate medical students, with patient-centered outcomes. The outcome measures were based on important elements of patient-centered care: patient satisfaction, self-care, health literacy, treatment compliance, and health attitudes, patient empowerment, student communication skills, shared decision-making, and relations between (future) care professionals and patients.

We removed duplicates automatically using EndNote (version 7.2; Clarivate Analytics, New York, NY, USA). Screening and inclusion of records was conducted independently by two researchers (TV, TB). We screened titles and abstracts to exclude records not describing both patient education and undergraduate medical students. Full-text assessment was performed to include studies that specifically studied a student-provided patient education intervention that targeted real patients and was aimed at the outcomes used in the search strategy. We discussed disagreements on screening and inclusion until agreement was reached. References of included studies were checked for other relevant studies.

We determined study quality using the Quality Assessment Tool for Quantitative Studies, which is specifically aimed at assessing the quality of public health studies.^20^ Two researchers (TV, TB) independently evaluated study quality and resolved disagreements through discussion in order to reach final study quality ratings. The tool guidelines state that any study with two or more weak scores on subcategories is considered to have a weak overall score. Since most of the studies in our review had low evidence levels and low study quality, we chose to modify the overall rating method by using an averaging of ratings on subcategories to be able to differentiate between ratings on study quality.

Included studies were characterized by one researcher (TV) with regard to intervention method, outcomes that were concordant with the outcomes of the search strategy, the subject of patient education, the patient target group, the educational stage of the medical students involved, the care setting of patient education, and the study location. The four-level Kirkpatrick model was used to categorize the level of effects (experiences, learning, behavior, and organizational impact) of the interventions on both patients and students.^21^

Two patients, two care professionals, and two researchers, all working in the field of patient empowerment and medical education, rated the studies on a scale of 1 to 10 in terms of their impact on quality of care and their practical feasibility. Five education professionals in medical education rated the studies on a scale of 1 to 10 on the basis of learning yield and practical feasibility. In addition, an overall score was determined by all experts based on study quality, intervention characteristics, and outcomes. Ratings were based on their individual experience and expertise in medical education and quality of care. To guide the rating process, the experts received a document describing the intervention methods, outcomes, and quality of the studies, as presented in Table 1, and a guideline which included the goal of rating the studies, a description of information presented in the table, and definitions of quality of care, learning yield, and practical feasibility. Higher- and lower-than-average scores were used to categorize and compare the interventions. The intra-class correlation coefficient of expert rating groups was calculated using SPSS software (version 22; IBM Corp., Armonk, NY, USA) in a two-way mixed model to determine rating consistency.

**Table 1:** Overview of Interventions and Outcomes of Student-Provided Patient Education

Study title	Study design, evidence level, and overall quality	Intervention method, students’ stage, patient education subjects, and patient target group	Number of participants [n(p) = number of patients, n(s) = number of medical students] and effect sizes (P1–4 = Kirkpatrick level of patient outcome, S1–4 = Kirkpatrick level of student outcome)
Standardized instructions: do they improve communication of discharge information from the emergency department?^22^	Study design: non-randomized controlled trial	Medical students providing verbal and written discharge instructions to parents of children with otitis media, after consultation with an attending physician	n(p) = 136 n(s) = not reported P1) Parent satisfaction: 96%
	Evidence level: 3		
	Study quality: moderate		
Health fairs as a unique teaching methodology^23^	Study design: post-intervention survey	First- and second-year medical students organizing a community-based health fair, with information about blood pressure, diabetes, and explanation of lab results to patients with hypertension, carcinomas, nipple retraction, or chronic infection	n(p) = 152 n(s) = 213 P1) Patient satisfaction: 93% of patients rated good or higher
	Evidence level: 4		
	Study quality: weak		
An office-based Internet patient education system: a pilot study^24^	Study design: post-intervention interview	Medical students assisting patients with use of the Internet/computer for patient education on the Web	n(p) = 50 n(s) = not reported P1) Patient satisfaction: 90% of patients more satisfied with visit to clinic than usual P3) Patient-reported change in behavior after patient education: 77% of patients
	Evidence level: 4		
	Study quality: weak		
The attitudes of cardiac arrest survivors and their family members towards CPR courses^25^	Study design: non-randomized controlled trial	Medical students providing courses in basic and advanced life support for cardiac arrest survivors and their families in comparison with general public (subjects: diagnosing unconsciousness, respiratory and cardiac arrest, and CPR)	n(p) = 101 n(s) = 9 P1) Patient satisfaction: 96% of patients rated good or higher P2) Patient-reported knowledge of CPR: 96% of patients understand principle after training P2) Patient-reported confidence in performing CPR after training: 79% of patients
	Evidence level: 3		
	Study quality: moderate		
Applying practical preventive skills in a preclinical preceptorship^26^	Study design: uncontrolled before-and-after study	Preclinical medical students providing foot-care education to diabetic patients during preclinical preceptorship	n(p) = 321 n(s) = 158 S2) Student-reported self-efficacy in patient education: 0% of students before vs. 90% of students after (significance not reported)
	Evidence level: 4		
	Study quality: moderate		
Evaluating a diabetes foot care program in a preceptorship for medical students^27^	Study design: post-intervention survey	Preclinical medical students providing foot-care education for diabetic patients during 4-week ambulatory educational experience	n(p) = 310 n(s) = 156 P1) Patient satisfaction: 90.3% of patients rated valuable P2) Patient-reported improved knowledge: 84% of patients P2) Patient-reported improved health attitude: 88.8% of patients
	Evidence level: 4		
	Study quality: weak		
The summer assistantship in patient education: a preclinical preceptorship^28^	Study design: post-intervention survey	Medical students between the first and second year educating and counseling people with arthritis, diabetes, depression, or hypertension in family practice full-time over 5–7 weeks in summer	n(p) = 6000 encounters n(s) = 40 S1) Student satisfaction: 98% of students very satisfied S2) Student-reported improved skills: 90% of students
	Evidence level: 4		
	Study quality: weak		
Enhancing the relationship and improving communication between adolescents and their health care providers: a school based intervention by medical students^29^	Study design: post-intervention survey	Second- and fourth-year medical students giving a workshop at high schools about communicating with professionals and legal/ethical aspects of care. Learners in the high school were children who visit the primary care physician	n(p) = 1651 n(s) = 181 P1) Patient satisfaction: 94% of patients scored workshop as “just right” P3) Patient-reported behavior on follow-up: 57% of patients experienced difference in encounter with physician (n(p) = 17)
	Evidence level: 4		
	Study quality: weak		
A wellness class for inpatients with psychotic disorders^30^	Study design: uncontrolled before-and-after study	Medical students giving 30-min didactic presentations about diet and exercise to inpatients with chronic psychotic disorders	n(p) = 50 n(s) = not reported P2) Knowledge of patients on exam: 4.3% improved score on exam before intervention and after intervention (*p* < 0.02)
	Evidence level: 4		
	Study quality: weak		
A preclinical training model for chronic care education^31^	Study design: post-intervention survey	Preclinical medical students counseling diabetic patients in ambulatory care about diabetes foot care	n(p) = 424 n(s) = 124 P1) Patient satisfaction: 95% of patients rated useful
	Evidence level: 4		
	Study quality: weak		
Making health literacy real: adult literacy and medical students teach each other^32^	Study design: post-intervention survey	Medical students giving presentations about health literacy (e.g., living with diabetes or controlling blood pressure) to adult learners who are following a literacy course and have hypertension, diabetes, cancer, depression, or mental illness	n(p) = 30 n(s) = 45 P2) Patient-reported improved knowledge: 44% of patients P2) Student-reported improved communication skills: 88% of students
	Evidence level: 4		
	Study quality: weak		
Caring for underserved patients through neighborhood health screening: outcomes of a longitudinal, interprofessional, student-run home visit program in Singapore^33^	Study design: uncontrolled before-and-after study	Different grades of medical students providing in-home medical services (e.g., information on disease management, medication/treatment compliance, managing complications) to patients with hypertension, diabetes, dyslipidemia, colorectal cancer, or cervical cancer in a low-income neighborhood	n(p) = 209 + 355 = 564 (two cohorts) n(s) = 240 P1) Patient satisfaction: 82% of patients satisfied (*n* = 291, cohort 2) P3) Blood pressure control: 42% hypertensive patients pre vs. 79% hypertensive patients post (*n* = 82, *p* < 0.001, cohort 1) S1) Student satisfaction: 70% of students S2) Student-reported development of communication skills: 98.6% of students S2) Student-reported improvement of relationship: 92.8% of students S2) Student-reported improved self-efficacy in patient counseling: 92.3% of students
	Evidence level: 4		
	Study quality: moderate		
Effects of interprofessional education on patient perceived quality of care^34^	Study design: non-randomized controlled trial	Fourth-year medical students participating in inter-professional student teams at clinical education ward and providing information on treatment, daily living with disease, and self-care	n(p) = 102 treatment vs. 85 control group n(s) = not reported In comparison with regular care P) Patient-reported involvement in decisions: 62% higher number of patients in clinical education ward P2) Patient-reported knowledge of daily living with disease: 50% higher number of patients in clinical education ward (*p* < 0.006) P2) Patient-reported understanding of treatment information: 69% higher number of patients in clinical education ward (*p* < 0.02)
	Evidence level: 3		
	Study quality: moderate		
The clinical skills experience of rural immersion medical students and traditional hospital placement students: a student perspective^35^	Study design: non-randomized controlled trial	Sixth-year medical students participating in rotations in rural practice and providing patient education to rural community	n(p) = not reported n(s) = 6 treatment vs. 17 control S2) Student-reported improved patient education self-efficacy compared to traditional rotations: 31.6% higher number of students confident in providing patient education (significance not reported)
	Evidence level: 3		
	Study quality: weak		
The crimson care collaborative: a student-faculty initiative to increase medical students’ early exposure to primary care^36^	Study design: case series	Student-designed and student-run clinic providing primary care services (such as patient education about medication or designing patient education materials) with preclinical and clinical medical students	n(p) = 17 n(s) = not reported P3) Blood pressure control in 76% of 17 patients who visited the clinic vs. 48.4% in average population (significance not reported)
	Evidence level: 4		
	Study quality: weak		
Approach to antihypertensive adherence: a feasibility study on the use of student health coaches for uninsured hypertensive adults^37^	Study design: uncontrolled before-and-after study	Clinical-year medical students’ health coaching for uninsured hypertensive patients of a free clinic, e.g., making phone calls to patients once every 2 weeks and explaining medication use, home blood pressure monitoring, and encouraging lifestyle goals	n(p) = 25 n(s) = 5 P1) Patient satisfaction: 92.8% of patients rated just right P2) Patient-reported improved knowledge: 71.4% of patients rated very much P3) Patient-reported improved behavior to hypertension: 92.9% of patients rated very much P3) Medication adherence on Brief Medication Questionnaire (BMQ) adherence scale: pre 2.33 vs. post 1.25, lower is better (*p* < 0.1) P3) Blood-pressure control: 147/92 average (SD = 18.7/17.4) pre-test vs. 136/85 average (SD = 18.3/6.5) post-test (systolic blood pressure shows significant difference with *p* < 0.03)
	Evidence level: 4		
	Study quality: weak		
Teaching patient-centered communication skills: a telephone follow-up curriculum for medical students^38^	Study design: non-randomized controlled trial	Third-year medical students in clinical rotation telephoning neurology, psychiatric, or surgical patients at home about medication adherence, comprehension of treatment plan, and understanding of illness, 1 week after clinical encounter	n(p) = not reported n(s) = 101 S1) Student-reported value/satisfaction: 84.2% of students S2) Student-reported deepening relationship with patients: 18.4% of students S2) Student-reported improved skills in patient education: 71% of students S3) Student-reported change in patient education behavior: 41% of students
	Evidence level: 3		
	Study quality: moderate		
Involving medical students in informed consent: a pilot study^39^	Study design: post-intervention interview	Sixth-year medical students providing additional conversation about surgery (surgical complications or risks) with surgical patients shortly before surgery	n(p) = 55 n(s) = 9 P2) Patient-reported improved understanding of treatment: 96.4% of patients S2) Student-reported improved self-efficacy in patient education: 100% of students
	Evidence level: 4		
	Study quality: weak		

Overview of interventions and outcomes reported in the included studies (*n* = 18). Column 2 shows the study design, evidence level, and overall quality. Column 3 shows the characteristics of the interventions: intervention method, students’ stage in medical education, patient education subjects, and patient target group. Column 4 shows the reported effects concordant with the outcomes used in the search strategy (patient satisfaction, self-care, health literacy, treatment compliance, health attitude, patient empowerment, students’ communication skills, shared decision-making, and relations between [future] care professionals and patients) and the reported effects on student satisfaction, self-efficacy in patient education, patient education skills, and patient education behavior, and on disease management

Finally, we used a customized assessment tool based on the Learning Transfer System Inventory (LTSI) to assess facilitators of and barriers to student-provided patient education. The LTSI is a validated model that describes factors influencing the transfer of a training intervention on the individual.^40^ Based on this model, we formulated facilitators and barriers in practice-based learning. Qualitative assessment of the reported facilitators and barriers was performed by two researchers (TV, CF) by coding study elements that were in concordance with the LTSI-based assessment tool using ATLAS.ti (version 7.1.5; Scientific Software Development GmbH, Berlin, Germany). Differences between coded facilitators and barriers were discussed to reach agreement.

## RESULTS

### Search Results

**Figure 1:**
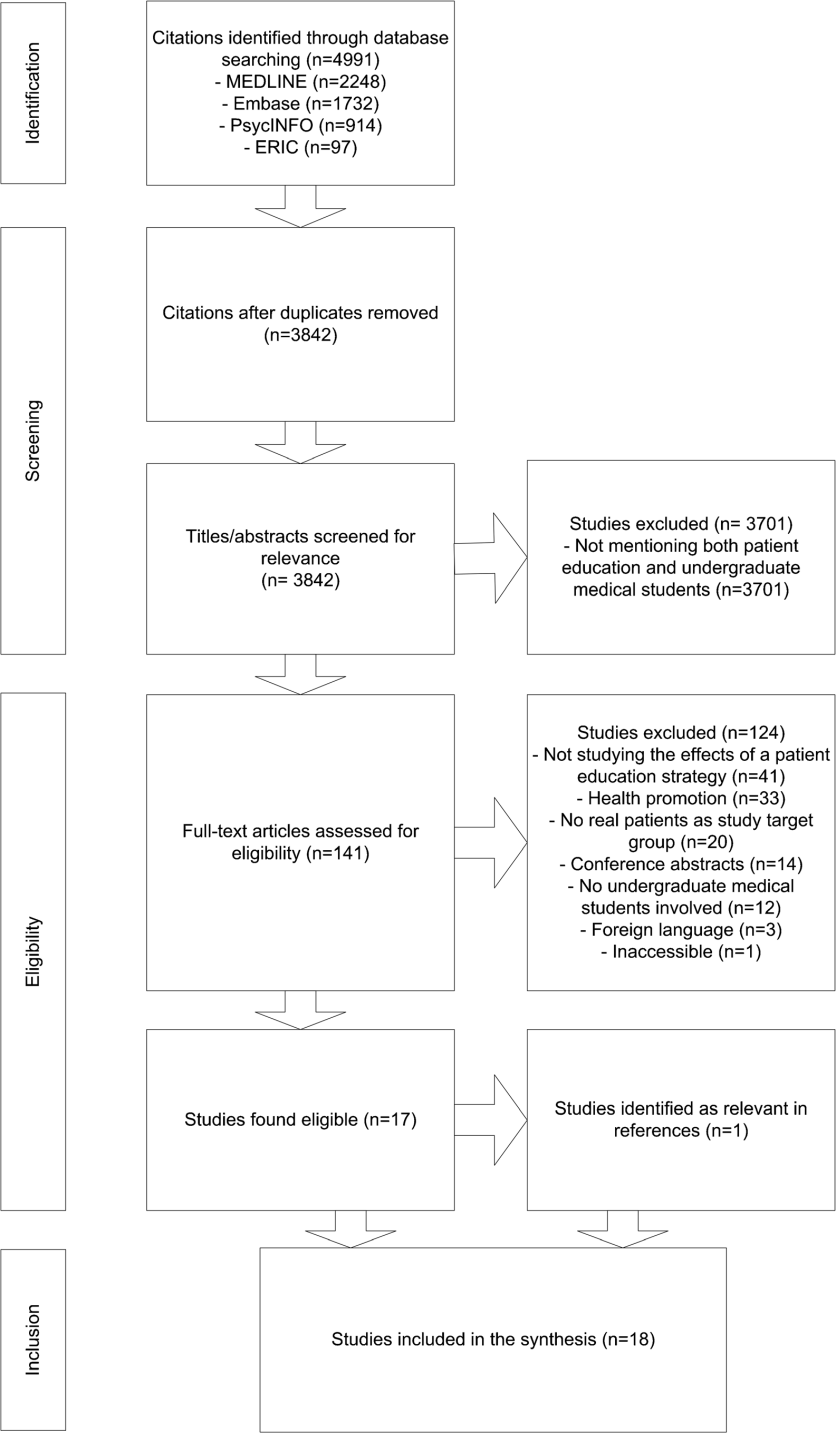
PRISMA flow diagram showing the number of records and studies identified during the search process, screened for relevance, assessed for eligibility and included in the synthesis. Reasons for exclusion of records or studies during the screening and eligibility process were categorized per group and are visualized per group and in total

The search resulted in 4991 records. After removing duplicates, 3842 titles and abstracts were screened for relevance, leading to the exclusion of 3701 studies. Full-text assessment was performed in 141 studies to determine eligibility, resulting in the inclusion of 17 studies in the final synthesis. A search of the references of the included studies revealed one additional relevant study, which was added to the synthesis (Fig. 1). In total, five non-randomized controlled trials,^22^^,^ 25 ^,^ 34 ^,^ 35 ^,^ 38 four uncontrolled before-and-after studies,^26^^,^ 30 ^,^ 33 ^,^ 37 eight post-intervention survey or interview studies,^23^^,^ 24 ^,^ 27 ^–^ 29 ^,^ 31 ^,^ 32 ^,^ 39 and one case series study^36^ were included.

### Interventions and Outcomes

Geographically, 12 studies were performed in the USA,^22^^–^ 24 ^,^ 26 ^–^ 28 ^,^ 30 ^–^ 32 ^,^ 36 ^–^ 38 three in the European Union,^25^^,^ 34 ^,^ 39 and one each in Canada,^29^ New Zealand,^35^ and Singapore.^33^

The studies described the following: medical students providing patient education during clerkships aimed at learning to provide patient education;^26^^–^ 28 ^,^ 31 ^,^ 35 medical students providing patient education courses or other types of teaching to patients and family members;^24^^,^ 25 ^,^ 29 ^,^ 30 ^,^ 32 medical students supporting patients in the context of treatment;^22^^,^ 38 ^,^ 39 medical students performing patient education to reach underserved communities;^23^^,^ 33 ^,^ 37 and medical students providing patient education in a student-run clinic or teaching clinic^34^^,^ 36 (Table 1).

The included studies involved undergraduate medical students at different stages of medical education. Several studies did not describe the medical students’ educational stage.^22^^,^ 24 ^,^ 25 ^,^ 30 ^,^ 32 ^,^ 33

Most involved student participation in providing patient education in primary care.^24^^,^ 26 ^–^ 28 ^,^ 31 ^,^ 36 ^,^ 37 Others involved medical students in the community,^23^^,^ 29 ^,^ 32 ^,^ 33 in the surgical department,^38^^,^ 39 the emergency department,^22^^,^ 25 in psychiatry,^30^^,^ 38 or in rural practice.^35^ Specifically, medical students provided patient education with regard to diabetes,^23^^,^ 26 ^–^ 28 ^,^ 31 ^–^ 33 hypertension,^23^^,^ 28 ^,^ 32 ^,^ 33 ^,^ 37 mental illnesses,^28^^,^ 32 arthritis,^28^ cardiac arrest,^25^ communicating with care professionals,^29^ treatment plans and options,^38^ surgical procedures,^39^ discharge instructions,^22^ use of digital tools,^24^ medication,^33^^,^ 36 disease-related lifestyle issues,^30^^,^ 32 ^,^ 37 and self-care.^34^

Nine studies reported on patient satisfaction.^22^^–^ 25 ^,^ 27 ^,^ 29 ^,^ 31 ^,^ 33 ^,^ 37 Aspects of patient-centered care were reported to be improved in student-provided patient education.^41^^,^ 42 Six studies reported increased self-reported health or disease knowledge,^25^^,^ 27 ^,^ 32 ^,^ 37 ^,^ 39 which was significant in one study (*p* < 0.006).^34^ One study reported enhanced health or disease knowledge (*p* < 0.02).^30^ One study reported improved self-reported confidence with regard to self-management.^25^ Another study reported improved shared decision-making.^34^ Two studies reported improved self-reported communication skills,^32^^,^ 33 and two reported improved student–patient relations.^33^^,^ 38

In terms of health-related outcomes, four studies reported a change in patients’ self-reported behavior or attitude toward their disease.^24^^,^ 27 ^,^ 29 ^,^ 37 Three studies described improved disease management^36^ (two studies with significant differences, *p* < 0.001 and *p* < 0.03, respectively).^33^^,^ 37 Another study reported improved self-reported medication adherence (*p* < 0.01).^37^

Student outcomes of student-provided patient education were described at Kirkpatrick levels 1–3. Three studies showed student satisfaction (level 1).^28^^,^ 33 ^,^ 38 Four studies reported enhanced self-reported patient education self-efficacy (level 2).^26^^,^ 33 ^,^ 35 ^,^ 39 Two studies reported positive effects in terms of improved self-reported patient education skills.^28^^,^ 38 In addition, two studies reported improved communication skills.^32^^,^ 33 One study described a self-reported change in students’ patient education behavior (level 3).^38^

### Study Quality Assessment

Six studies had moderate scores on study quality,^22^^,^ 25 ^,^ 26 ^,^ 33 ^,^ 34 ^,^ 38 and 12 studies had weak scores^23^^,^ 24 ^,^ 27 ^–^ 32 ^,^ 35 ^–^ 37 ^,^ 39 (Table 2). Weak quality ratings were the result of the following factors: uncertainty about representative participants in six studies;^23^^,^ 28 ^,^ 30 ^,^ 32 ^,^ 35 ^,^ 37 less than 60% participation among selected individuals in four studies^23^^,^ 25 ^,^ 32 ^,^ 35; weak study design in nine studies;^23^^,^ 24 ^,^ 27 ^–^ 29 ^,^ 31 ^,^ 32 ^,^ 36 ^,^ 39 study participant characteristics were not investigated in depth or compared to the general population in 15 studies;^23^^,^ 24 ^,^ 26 ^–^ 33 ^,^ 35 ^–^ 39 important baseline differences between groups in two studies;^35^^,^ 38 no reported use of valid or reliable measurement tools in 16 studies;^22^^–^ 33 ^,^ 35 ^,^ 36 ^,^ 38 ^,^ 39 and no reports of withdrawals or dropouts in three studies.^25^^,^ 35 ^,^ 38 In total, four non-randomized controlled trials^22^^,^ 25 ^,^ 34 ^,^ 38 and two uncontrolled before-and-after studies^26^^,^ 33 had moderate scores on study quality.

**Table 2:** Study Quality Overview

Study title	Selection bias	Study design	Confounders	Blinding	Data collection	Withdrawals	Overall study quality
Standardized instructions: do they improve communication of discharge information from the emergency department?^22^	2	1	1	2	3	1	Moderate
Health fairs as a unique teaching methodology^23^	3	3	3	2	3	2	Weak
An office-based Internet patient education system: a pilot study^24^	2	3	3	2	3	2	Weak
The attitudes of cardiac arrest survivors and their family members towards CPR courses^25^	3	1	1	2	3	3	Moderate
Applying practical preventive skills in a preclinical preceptorship^26^	2	2	3	2	3	1	Moderate
Evaluating a diabetes foot care program in a preceptorship for medical students^27^	2	3	3	2	3	2	Weak
The summer assistantship in patient education: a preclinical preceptorship^28^	3	3	3	2	3	2	Weak
Enhancing the relationship and improving communication between adolescents and their health care providers: a school based intervention by medical students^29^	2	3	3	2	3	2	Weak
A wellness class for inpatients with psychotic disorders^30^	3	2	3	2	3	2	Weak
A preclinical training model for chronic care education^31^	2	3	3	2	3	2	Weak
Making health literacy real: adult literacy and medical students teach each other^32^	3	3	3	2	3	2	Weak
Caring for underserved patients through neighborhood health screening: outcomes of a longitudinal, interprofessional, student-run home visit program in Singapore^33^	1	2	3	2	3	1	Moderate
Effects of interprofessional education on patient perceived quality of care^34^	2	1	1	2	1	2	Moderate
The clinical skills experience of rural immersion medical students and traditional hospital placement students: a student perspective^35^	3	1	3	2	3	3	Weak
The crimson care collaborative: a student-faculty initiative to increase medical students’ early exposure to primary care^36^	3	3	3	2	3	1	Weak
Approach to antihypertensive adherence: a feasibility study on the use of student health coaches for uninsured hypertensive adults^37^	3	2	3	2	2	3	Weak
Teaching patient-centered communication skills: a telephone follow-up curriculum for medical students^38^	2	1	3	2	3	3	Moderate
Involving medical students in informed consent: a pilot study^39^	2	3	3	2	3	2	Weak

Overview of quality assessment for included studies (*n* = 18). Studies were rated on a scale of 1 to 3 (1 = strong, 2 = moderate, 3 = weak), according to the Quality Assessment Tool for Quantitative Studies, on selection bias, study design, confounders, blinding, data collection, and withdrawals. Overall scores were determined as the average of all ratings, rounded up to whole numbers

### Expert Ratings

Patients, care professionals, and researchers in the field of patient empowerment expected that various interventions would have a higher-than-average impact on quality of care,^22^^,^ 25 ^,^ 27 ^–^ 29 ^,^ 32 ^–^ 34 ^,^ 36 ^,^ 37 whereas eight studies were expected to have a lower-than-average impact on quality of care. Education professionals rated seven studies with higher-than-average learning yield^23^^,^ 25 ^,^ 28 ^,^ 33 ^,^ 34 ^,^ 36 ^,^ 37 and 11 studies with lower-than-average learning yield. All experts rated seven studies with a higher-than-average overall score,^24^^,^ 25 ^,^ 28 ^,^ 33 ^,^ 34 ^,^ 36 ^,^ 37 and 11 studies with a lower-than-average overall score (Table 3).

**Table 3:** Expert Ratings on Interventions and Outcomes of Student-Provided Patient Education

Study title	Learning yield [education professionals *n* = 5, mean (SD)]	Impact on quality of care [patients *n* = 2, care professionals *n* = 2, researchers *n* = 2, mean (SD)]	Practical feasibility [all stakeholders *n* = 11, mean (SD)]	Overall score [all stakeholders *n* = 11, mean (SD)]
Higher-than-average scores on all aspects				
The attitudes of cardiac arrest survivors and their family members towards CPR courses^25^	7.6 (0.9) ^†^	7.3 (1.4) ^†^	7.8 (1.2) ^†^	7.8 (0.6) ^†^
Higher-than-average learning yield, impact on quality of care, and overall score				
Effects of interprofessional education on patient perceived quality of care^34^	8.4 (0.5) ^†^	7.3 (0.8) ^†^	6.7 (1.0)	7.8 (0.3) ^†^
The crimson care collaborative: a student–faculty initiative to increase medical students’ early exposure to primary care^36^	8.0 (1.0) ^†^	7.7 (1.4) ^†^	6.9 (1.6)	7.7 (1.0) ^†^
The summer assistantship in patient education: a preclinical preceptorship^28^	8.2 (0.4) ^†^	7.7 (1.0) ^†^	6.8 (1.8)	7.6 (1.1) ^†^
Caring for underserved patients through neighborhood health screening: outcomes of a longitudinal, interprofessional, student-run home visit program in Singapore^33^	8.4 (0.5) ^†^	8.2 (1.2) ^†^	6.1 (2.0)	7.4 (1.7) ^†^
Approach to antihypertensive adherence: a feasibility study on the use of student health coaches for uninsured hypertensive adults^37^	7.4 (0.5) ^†^	7.7 (0.5) ^†^	6.8 (1.2)	7.1 (0.7) ^†^
Higher-than-average impact on quality of care				
Standardized instructions: do they improve communication of discharge information from the emergency department?^22^	6.0 (1.0)	7.0 (1.7) ^†^	8.3 (1.0) ^†^	7.0 (1.0)
Evaluating a diabetes foot care program in a preceptorship for medical students^27^	6.6 (1.1)	7.0 (1.1) ^†^	7.0 (0.6)	7.0 (0.8)
Making health literacy real: adult literacy and medical students teach each other^32^	6.6 (1.1)	7.5 (1.6) ^†^	6.8 (1.2)	6.9 (1.3)
Enhancing the relationship and improving communication between adolescents and their health care providers: a school based intervention by medical students^29^	5.6 (1.3)	6.8 (0.8) ^†^	6.8 (0.6)	6.5 (1.0)
Higher-than-average learning yield				
Health fairs as a unique teaching methodology^23^	7.4 (1.1) ^†^	5.2 (2.3)	7.1 (1.2)	6.4 (1.7)
Higher-than-average practical feasibility				
An office-based Internet patient education system: a pilot study^24^	6.6 (1.1)	6.5 (2.0)	7.3 (1.0) ^†^	7.1 (0.8) ^†^
Involving medical students in informed consent: a pilot study^39^	6.8 (0.4)	5.7 (2.2)	7.4 (1.4) ^†^	6.8 (1.1)
Applying practical preventive skills in a preclinical preceptorship^26^	7.0 (0.7)	6.2 (1.2)	7.2 (0.8) ^†^	6.8 (0.9)
Teaching patient-centered communication skills: a telephone follow-up curriculum for medical students^38^	6.8 (1.6)	6.2 (2.1)	7.3 (0.8) ^†^	6.6 (1.4)
Lower-than-average scores on all aspects				
A preclinical training model for chronic care education^31^	6.2 (1.1)	6.7 (0.8)	6.9 (0.9)	6.9 (0.7)
The clinical skills experience of rural immersion medical students and traditional hospital placement students: a student perspective^35^	6.4 (0.5)	6.3 (1.5)	6.5 (1.4)	6.2 (1.0)
A wellness class for inpatients with psychotic disorders^30^	5.8 (1.5)	5.7 (1.0)	6.9 (0.8)	5.8 (1.1)
Average of all scores*	7.0	6.7	7.1	7.0
Intra-class correlation coefficient^‡^	0.79^§^	0.54^§^	0.51	0.71^§^

Overview of expert ratings on interventions and outcomes as reported in the included studies (*n* = 18). Five education professionals rated the learning yield, practical feasibility, and overall score. Two patients, two care professionals, and two researchers in the field of patient education and medical education rated the impact on quality of care, practical feasibility, and overall score. Ratings on practical feasibility and overall score were combined between expert groups. Mean and standard deviations of the ratings on all aspects are shown per study

*Average of all scores was calculated to enable comparison between interventions^†^Higher-than-average scores were used for categorization and comparison^‡^The intra-class correlation coefficient was determined using a two-way mixed model to determine consistency among ratings on each aspect

^§^Significant consistency (*P* < 0.05, F-test) was found between ratings of impact on quality of care, learning yield, and overall score

Only one intervention, which involved medical students providing cardiac arrest courses to patients and family members, received high ratings on learning yield, quality of care, and practical feasibility.^25^ Five interventions—student-provided clinics and programs for diverse patient groups, a summer clerkship aimed at patient education, and student health coaching for uninsured patients—were given high ratings on learning yield and impact on quality of care, but were rated as having low practical feasibility.^28^^,^ 33 ^,^ 34 ^,^ 36 ^,^ 37 One intervention in which students provided discharge instructions was rated as having a high impact on quality of care and practical feasibility, but low learning yield.^22^ In addition, three interventions—providing diabetes foot-care education during a preceptorship, a student-provided course on health literacy, and a student-provided course on communication with physicians—were rated as having a high impact on quality of care, low learning yield, and low practical feasibility.^27^^,^ 29 ^,^ 32 One intervention, involving a student-provided patient education health fair, was rated as having a higher learning yield but low impact on quality of care and practical feasibility.^23^ Low learning yield, low impact on quality of care, and high practical feasibility were found in four interventions: students providing follow-up telephone calls after discharge, providing enhanced communication with patients regarding informed consent for surgery, assisting patients in using the Internet for patient education, and providing diabetes foot-care education.^24^^,^ 26 ^,^ 38 ^,^ 39 Three interventions had low ratings on all aspects: students providing wellness classes for inpatients with psychotic disorders, students providing diabetes foot-care education, and students providing patient education in the rural community.^30^^,^ 31 ^,^ 35

The consistency among ratings of (1) education professionals on learning yield; (2) patients, care professionals, and researchers on impact on quality of care; and 3) all stakeholders on overall score was 0.548–0.795, and was significant (*P* < 0.05). The intra-class correlation coefficient of expert ratings on the practical feasibility of the interventions, on the contrary, was 0.511 and was non-significant.

### Facilitators and Barriers

An in-depth assessment of the studies showed that in most interventions, students were prepared through orientation or training sessions before their practical experience with real patients.^23^^,^ 25 ^–^ 29 ^,^ 31 ^,^ 37 ^–^ 39 Written or oral feedback or support provided by supervisors, fellow students, or patients were also reported to facilitate the effectiveness of patient education.^26^^,^ 27 ^,^ 31 ^–^ 36 ^,^ 39 Peer support from other students was provided in most interventions, at the individual or organizational level, facilitating the students’ learning achievements.^23^^,^ 29 ^,^ 33 ^,^ 34 ^,^ 36 Various learning aids, such as leaflets, were provided to students to enhance learning opportunities.^26^^,^ 27 ^,^ 31 ^,^ 37 A transfer design approach was applied in most studies, in which the training program resembled the future job and the students were part of the treatment team in a professional role, which facilitated learning effectiveness^23^^,^ 26 ^–^ 29 ^,^ 32 ^–^ 34 ^,^ 36 ^,^ 37 ^,^ 39 (Table 4).

**Table 4:** Overview of Facilitators and Barriers in Educational Interventions on Student-Provided Patient Education

Category	Facilitators	Barriers
Trainee characteristics		
Learner readiness	- Orientation or training sessions prior to performing patient education, consisting of theoretical and practical fundamentals for providing patient education^23^^,^ 25 ^–^ 29 ^,^ 31 ^,^ 37 ^–^ 39	None reported
Performance self-efficacy	- Students recognizing their independence in helping patients^26^ - Students recognizing their skills after talks with supervisors^31^^,^ 39 - Students recognizing their skills in patient education after feedback from patients^33^	- Students having the feeling during the training that they were not capable of contributing to patient education^38^
Motivation scales		
Motivation to learn	- Preselected students based on exam results are more motivated^28^ - Voluntary application to participate in the study includes highly motivated students^23^^,^ 33	- Nature of student participation too voluntary, giving them the feeling that participation was not important^26^ - Strategy not applicable to all students because of voluntary application of only highly motivated students^33^
Transfer effort-Performance expectations	- Students’ perception that training effort leads to better skills in patient education^23^^,^ 28 ^,^ 29 ^,^ 32 ^,^ 38 ^,^ 39	- Students not recognizing their training effort as useful for enhancing their professional role^38^
Performance-Outcomes expectations	- Students recognizing that their contribution leads to better patient care^33^	- Students not seeing the importance of their contribution to patient care^38^
Environment scales		
Feedback/Performance coaching	- Feedback on performance by supervisor(s) in written or oral form (in presentation meetings or individually)^26^^,^ 28 ^,^ 31 ^,^ 33 ^,^ 34 ^,^ 36 ^,^ 38 ^,^ 39 - Feedback from fellow students^34^ - Feedback from patients on postcards or oral^27^	None reported
Supervisor support	- Practical supervision when performing patient education^26^^,^ 31 ^–^ 34 - Supervision as needed after patient education^27^^,^ 34 ^,^ 36 ^,^ 39 - Supervision from different professions^32^^,^ 34 ^,^ 35	- Lack of time for support from or supervision by preceptors^26^
Supervisor sanctions	None reported	- Supervisor(s) not acknowledging the importance of learning patient education^26^ - Students not enabled to perform patient education because their knowledge and skills are not recognized^26^
Peer support	- Students supporting student-provided patient education on organizational level^23^^,^ 29 ^,^ 33 ^,^ 36 - Different stages of medical students working together in teams^23^^,^ 33 - Senior medical students supervising or mentoring junior medical students^23^ - Interprofessional teams of students working together in providing patient education^34^	None reported
Resistance/Openness to change	- Voluntary application includes students who are open to learning and changing their behavior^23^^,^ 38	- Differences between male and female students in openness to changing their behavior^33^
Positive personal outcomes	- Appreciation of students by patients^26^^,^ 33 - Students feeling proud of having responsibility^23^^,^ 26 - Students having clearer vision of ambitions as a result of experiences^33^ - Students receiving personal tokens of appreciation from faculty^29^^,^ 32 - Students being appreciated by other students^33^	None reported
Ability scales		
Opportunity to learn	- Learning aids to assist students in providing patient education (e.g., leaflets)^26^^,^ 27 ^,^ 31 ^,^ 37 - Making students members of the team^28^ - Technical resources such as access to health records or laptops for ambulatory care^33^ - Additional funding to create initiatives^36^	None reported
Personal capacity for learning	- Enough time to perform patient education^29^ - Students adequately prepared to perform patient education^26^	- Time limitations due to other curricular activities^37^ - Workload too high or schedule too busy during preceptorships to perform patient education^38^
Perceived content validity	- Students appreciating and recognizing their role as physicians in performing patient education^33^ - Students recognizing training of various skills as preparing them for future work^29^^,^ 33 ^,^ 38 - Students appreciating experience with different patient perspectives to prepare them for their future job as physicians^29^^,^ 33 ^,^ 37	- Students not appreciated as team members, but as assistants^26^
Transfer design	- Making students part of the team to enhance learning^28^ - Focusing on students’ professional role in designing educational set-up, e.g., with regard to patient interaction, communication skills, responsibility^23^^,^ 26 ^,^ 27 ^,^ 29 ^,^ 32 ^,^ 34 ^,^ 37 ^,^ 39 - Providing students with patient care experiences to enable them to shape their future careers^26^^,^ 33 ^,^ 36 ^,^ 37 - Enabling students to work together with other professions in healthcare^34^ - Enabling students to perform medical roles independently^28^^,^ 29 ^,^ 34	None reported

Overview of facilitators and barriers in educational interventions on student-provided patient education. Categories and subcategories of the Learning Transfer System Inventory are shown in the first column. The second and third columns show the respective facilitators and barriers to educational interventions in each subcategory as reported in the studies

Preselecting motivated students, e.g., by voluntary application to the learning module or course, was discussed in the studies as being both a facilitator and barrier: motivated students were expected to perform better;^23^^,^ 28 ^,^ 33 students who regarded the training program as too voluntary, on the contrary, were reported to perform less well.^26^^,^ 33

One study reported that students felt they had not been able to contribute to patient care by providing patient education.^38^ Another mentioned that supervisors did not recognize the students’ skills in patient education or did not acknowledge the importance of students performing or practicing patient education.^26^ Yet another study reported that students did not have enough time to practice patient education on real patients, over and above other curricular activities.^37^

## DISCUSSION

Our findings suggest that involving undergraduate medical students in patient education has the potential to improve the quality of care and medical education. The included studies reported that student-provided patient education enhanced patient health or disease knowledge, health attitude, health behavior, medication adherence, disease management, and shared decision-making. In addition, enabling students to provide patient education was reported to enhance students’ patient education skills, patient education self-efficacy, patient education behavior, relations with patients, and communication skills. These findings support evidence that students greatly appreciate and benefit from practice-based patient interaction.^14^^,^ 43

Student-run patient education clinics, student-provided outreach programs, student health coaching, and clerkships on patient education, in particular, were rated by experts as having a higher-than-average learning yield and impact on quality of care, and thus should be implemented to improve the quality of care and medical education.^28^^,^ 33 ^,^ 34 ^,^ 36 ^,^ 37

The World Health Organization has defined six dimensions of quality of care: effectiveness, efficacy, accessibility, patient-centeredness, equity, and safety.^44^ The current review indicates that students can contribute to effective, accessible, and equitable healthcare. The interventions also led to improvements in important contributors to patient-centeredness of care, including patients’ health knowledge, self-management, shared decision-making, communication skills of (future) care professionals, and relations between patients and students.^41^^,^ 42 In addition, combining student-provided patient education with medical education can enhance the efficiency of care and medical education.

From a student perspective, student-provided patient education can enhance students’ self-efficacy in patient encounters in general, and it enables students to recognize their independence in assisting patients and can help them feel that they are capable of contributing to patient care. Peer support and collaboration among different levels of students can enhance teamwork skills and facilitate the development of other skills relevant for physicians, such as leadership and coaching. Moreover, student-provided patient education can provide students with further insight regarding their career perspective.

Medical students should be prepared for providing patient education in practice through the use of training or orientation sessions to improve the quality of the education they provide. Involving peers, preceptors or other supervisors, and patients in supervision or provision of feedback to students enhances their self-efficacy and gives them personal recognition for contributing to patient care.^45^ In line with the practice of workplace pedagogy, students in clinically embedded approaches should be included in the treatment team as equal members in order to enhance their independence and value in providing care.^46^ Finally, student peer support, such as the involvement of students from different stages of medical education, contributes to training effectiveness, for example, by improving students’ teamwork skills.^33^

Despite the high impact on quality of care and medical education, the practical feasibility of more complex interventions, such as student-run patient education clinics, outreach programs, student health coaching, and clerkships on patient education, was rated low by experts. Other interventions, such as medical student involvement in providing courses on cardiac or respiratory arrest,^25^ communication with doctors,^29^ or health literacy,^32^ or students providing discharge instructions to patients,^22^ may be practically more feasible, and were rated as having a high impact on quality of care. Other ratings by experts on practical feasibility in this review can be used to guide future practices and research in the field of medical education.

### Limitations

As an important aspect of medical education focuses on specialized care, we configured our search strategy for patient education studies (defined as educating or counseling people with a disease) rather than health promotion studies (defined as preventive education for the general public). Though we found records addressing health promotion performed by medical students, we excluded studies that did not address disease-related issues.

The search strategy used in this study was aimed at patient-centered outcomes of patient education. Improving health status with patient education is a subject of debate.^47^ Given that our review shows that disease management is enhanced with student-provided patient education, other studies may show that health status is improved as well.^48^

Finally, since our search was limited to the scientific literature, examples of integrating patient education and medical education as reported in the gray literature are not described in this review.

### Future Research

In light of the low to moderate quality of the studies included in this review, future research should examine the effects of student-provided patient education with high-level evidence, such as randomized controlled trials. Specifically, the evidence level was low in studies on health-related outcomes of student-provided patient education, such as patients’ disease attitude, medication adherence, and disease management, making them difficult to interpret. Future research should examine these impacts in high-quality studies.

In addition, since most outcomes in the selected studies were self-reported, social desirability bias may have influenced the results; future research should use validated and reliable methods such as observational research, (focus group) interviews, or knowledge and attitude questionnaires to improve the validity of effect evaluations.

Though other studies reported improved patient outcomes with student-provided patient education, high-quality studies were largely aimed at examining the impact on the quality of medical education. Since our findings suggest that both patients and students benefit from student-provided patient education, future studies should simultaneously assess these effects on both patients and students.

In the evaluations of effects in the current review, the greatest emphasis was placed on learner satisfaction and learning goals, such as obtaining knowledge or changing attitudes. Future research should also investigate the effects on students’ patient education behavior. Moreover, although it is expected that student-provided patient education can impact the practice of care and medical education, the impact at an organizational level was not investigated; such effects should be examined in future studies. In addition, various other effects of student-provided patient education were described, such as improved leadership skills and role independence, or enhanced career perspectives. Future studies should further investigate these effects on students.

Finally, most studies performed a before-and-after evaluation and missed the opportunity to examine whether the effects were sustained. Future studies should examine the longer-term effects on patients and students.

## CONCLUSIONS

The integration of patient education into medical education has the potential to improve quality of care and enhance medical education. In particular, student-run patient education clinics, student-provided outreach programs, student health coaching, and clerkships on patient education can contribute to quality of care and medical education and should be implemented in care practice and medical education.

Our review provides an extensive overview of ways that student-provided patient education can benefit quality of care and medical education. Given the low to moderate quality of the studies reviewed, further research is needed on the effects of student-provided patient education. Such future studies should (1) provide high-quality evidence of the effects on both patients and students; (2) further examine effects such as the impact on leadership skills, role independence, and career perspectives among students; 3) investigate the long-term effects on patients and students; 4) examine the impact on clinical and educational practice; and 5) further investigate the effects on health-related outcomes.
